# Impact of COVID-19 pandemic in children using non-invasive ventilation: a thematic analysis of caregivers answers to a survey study

**DOI:** 10.3389/frsle.2023.1328558

**Published:** 2024-01-10

**Authors:** Lauren Dobson, Ella Milne, Heather Halperin, Deborah Olmstead, Shannon D. Scott, Maria L. Castro-Codesal

**Affiliations:** ^1^Faculty of Nursing, University of Alberta, Edmonton, AB, Canada; ^2^Department of Pediatrics, University of Alberta, Edmonton, AB, Canada

**Keywords:** non-invasive ventilation, COVID-19 pandemic, child, CPAP, BPAP

## Abstract

**Purpose:**

The COVID-19 pandemic has resulted in drastic changes in people's lives, more so in individuals with chronic conditions, such as children with chronic respiratory disorders requiring home non-invasive ventilation. Our research question was: How has the COVID-19 pandemic affected the daily lives of children using home NIV and their families and their NIV adherence?

**Methods:**

An anonymous online survey was administered to caregivers of pediatric patients using home NIV followed at the Stollery's Pediatric NIV Program in Alberta, Canada, between September 2020 and September 2021. Thematic analysis was conducted for the identification of emerging themes.

**Results/findings:**

Four themes were identified: (1) positive effects, (2) negative effects, (3) neutral effects, and (4) impact on NIV adherence. Effects of COVID-19 on children and families were reported by 55 respondents (57% response rate). Positive effects included a slower lifestyle, more family time, and less recurrent acute respiratory illness. Negative effects included increased parental anxiety, prolonged social isolation beyond imposed restrictions, and limited access to health supplies. Despite these negative effects, 90% of respondents reported adequate maintenance or even increases in their child's NIV use. A general sense of benefit in the virtual specialized care model was also highlighted.

**Conclusion:**

COVID-19 resulted in varying levels of impact on the lives of children using NIV, not unlike the general population. Negative effects, however, appeared to intensify in these technology-dependent children. NIV adherence, however, was prioritized by families and even increased during COVID-19. Further research is needed to analyse the potential benefits of virtual models of specialized care.

## Introduction

In 2019 the novel coronavirus (COVID-19) began to impact lives worldwide as the highly contagious disease quickly spread into a global pandemic (Viola and Nunes, 2022). Literature shows that isolation connected to extensive COVID-19-based quarantine and physical distancing imposed by governments and health officials resulted in increased anxiety, depression, frustration, and behavioral challenges in both children and adults (Daly and Robinson, 2021; Mulugeta et al., 2021; Viola and Nunes, 2022). Further, previous reports demonstrated the potential of the effects of the pandemic to predispose, precipitate, and perpetuate sleep disturbances. This was cause for concern as sleep is critical for both physical and mental health, especially for the developing child (Cox and Olatunji, 2021). In addition to adapting to the pandemic, pediatric patients with chronic respiratory diseases, as other patients with medical complexity, were faced with the intimidating task of simultaneously managing the use of daily therapies and medical technology. This is the case for children using nocturnal non-invasive ventilation (NIV) therapies at home, either for airway patency through a continuous positive airway pressure (CPAP) or for lung recruitment and/or ventilation support through bilevel positive airway pressure (BPAP) (Ramirez et al., 2013). Recent research analyzing NIV machine download data in children between the initial 3 months and 12 months of the pandemic has shown overall high NIV use, although children struggling to use NIV before the pandemic continued to do so even further (Sunkonkit et al., 2022; Halperin et al., 2023). No prior research, however, has analyzed the lived experiences of children using home NIV therapies and their families during a pandemic and specifically the challenges in adhering to NIV.

This study aims to: (1) understand how the COVID-19 pandemic has affected the overall daily lives of children using home NIV and their families and (2) to gather the caregivers' understanding of how the COVID-19 pandemic has affected NIV adherence in these children.

## Methods

This study is a thematic analysis of written survey responses related to the impact of the COVID-19 pandemic on the lives of children from caregivers of children using home NIV, approved by the University of Alberta's Health Research Ethics Board.

In March 2020, Stollery's Pediatric NIV Program underwent a rapid transition to a fully virtual model of care. During the study period, clinical care, assessment of remote NIV machine download data and support for mask and headgear re-assessment were exclusively virtual. If headgear required adaptation, the family was booked with an occupational therapist for adaptation of interface headgear without the in-person presence of the patient. Regular visits every 6–18 months were maintained virtually as per our standard of practice.

### Sample

Participants were prospectively recruited through the Pediatric Home NIV Program at the Stollery Children's Hospital, Edmonton, Canada, during the period between September 2020 and September 2021. Inclusion criteria included regular caregivers of children 0–18 years who have been using home CPAP or BPAP for >3 months and who attended a regular visit with the program during the study period.

### Data collection and storage

Study participants were recruited upon completion of their clinic visit and provided with an anonymous online survey link to consent and complete the survey (completion time <10 min). Participants requiring translation were assigned an independent translator to read the questions by phone and were allowed to answer in their language and further translated to English. Participants who consented but did not complete the survey received two automatic reminders.

The survey posed two free-text questions:

- How has the COVID-19 pandemic affected your child's and your family's daily life? Please include challenges or stressors (ex. fears, financial changes, etc.) as well as any benefits or improvements (ex. less illness, better sleep, more family time, etc.) for your family.
- How has the current COVID-19 pandemic affected your child's NIV use?

Answers were stored in a secured REDcap database (Harris et al., 2009) along with anonymous demographic information for participating caregivers, and child's demographic and clinical information.

### Data management and analysis

Informed by Braun and Clarke's (2006) approach, thematic analysis methods were applied for data synthesis by two researchers (EM and LD). NVivo 12 (related to March 2020) was used for data management (QSR International Pty Ltd, 2018). The initial analysis included consistent coding data from the survey responses. Common patterns were identified, and then a deductive approach was used to group similar codes to develop relevant, emerging themes. A theme was defined as “an abstract entity that brings meaning and identity to a recurrent experience and its variant manifestations, capturing and unifying the nature of the experience into a meaningful whole,” based on Nowell et al. (2017) definition. Themes and subthemes reflective of the data were used to synthesize the data in a meaningful way. Emerging themes were discussed until a consensus was reached. Demographic data was summarized using proportions.

## Results

Out of 95 families offered the survey upon their clinic appointments, 56 responses were received (59% response rate). Most respondents were the child's mother (83%). Obstructive sleep apnea was the most reported indication for NIV therapy (Table 1). Other indications included complex respiratory needs due to a syndrome, a neuromuscular, respiratory, or neurological condition. Among these children, CPAP was more commonly used.

**Table 1:** Caregiver-reported demographic and clinical characteristics of their child using home NIV.

**Characteristic**	**%**
**Number of years using NIV**	
<1 year	11
1–5 years	63
>5 years	21
Not reported	5
**Therapy type**	
CPAP	59
BPAP	41
**Underlying diagnosis**	
Obstructive sleep apnea/other upper airway disorder	55
Down syndrome	11
A neuromuscular disease	18
Chronic lung disease	5
Neurological disease	9
Cardiac disease	0
Other (unknown)	2

**Figure 1:**
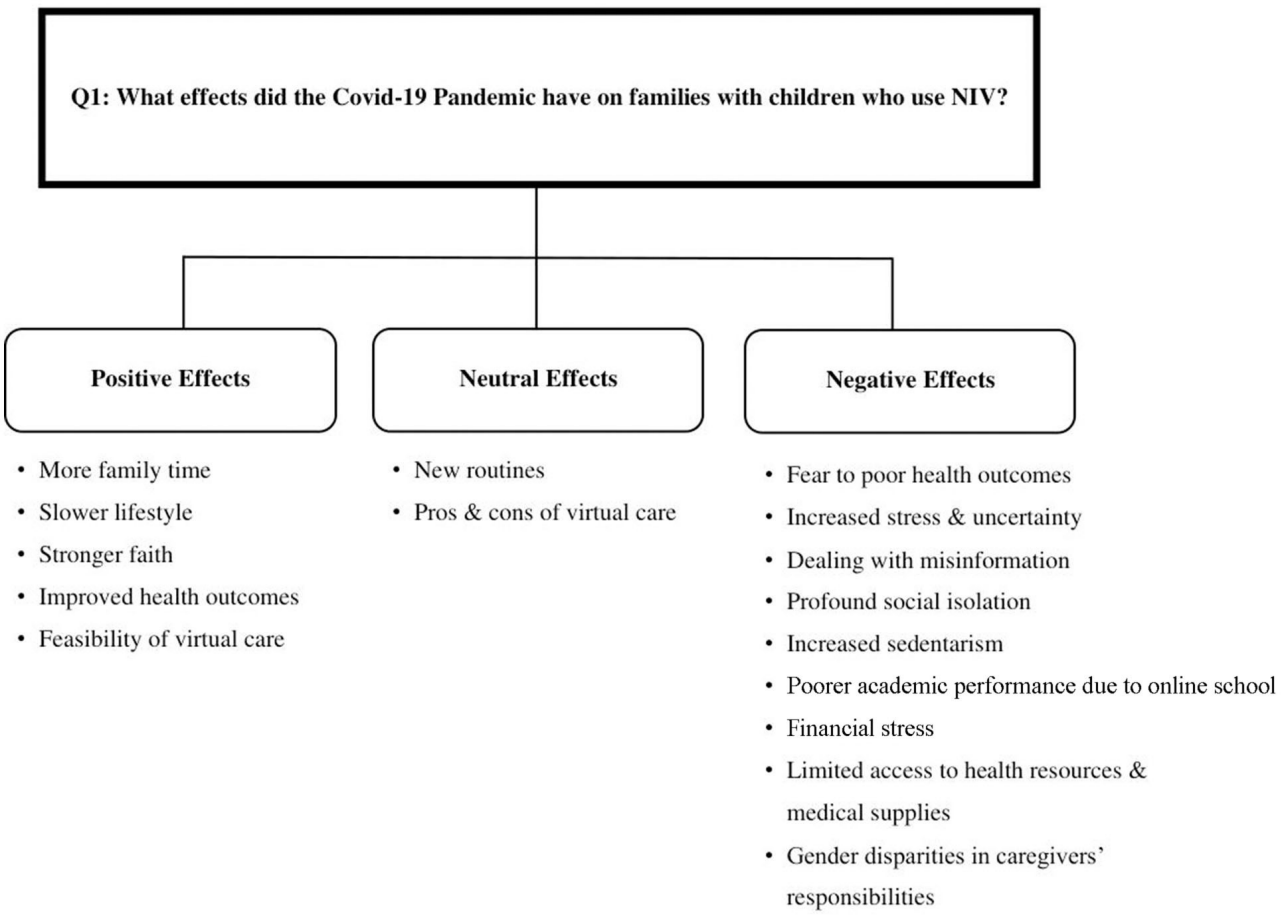
Caregiver-reported effects of COVID-19 pandemic on their child (NIV user) and family.

Four themes were identified in the data: (1) positive effects, (2) negative effects, (3) neutral effects, and (4) impact on NIV adherence (Figure 1).

### Theme 1: positive effects of COVID-19 on the families of children using NIV

Among positive outcomes, the most notable were having more family time, a slower lifestyle, and less illness (Table 2).

**Table 2:** Data excerpts for theme “positive effects of COVID-19 pandemic on child and family.”

“*When we were doing school at home, my child had more time to sleep in the morning that improved his mood, his attention and energy.”*—Participant 4
“*I think it was great that she was able to sleep in and not have to get up before her body naturally wanted to and that we were able to construct our days as we would in the summer months; not be dictated by a pre-set schedule (school, etc).”*—Participant 9
“*But we spend time to play board games and read [the] Bible and share thanks every day. Now we are blessed to stay together such as less illness, better sleep, more time hiking.”*—Participant 11
“*Less illness for us as a family on the whole. Husband is now working from home so is able to help out after work more with the kids now.”*—Participant 24
“*The upside to it all is that neither one of our children have gotten sick. Especially our older daughter, when she gets sick, she gets really sick and typically ends up in hospital. So, it has been nice that we all have been healthy.”*—Participant 15

Many participants conveyed that the COVID-19 pandemic allowed families to enjoy more time together, largely because of a newly slower lifestyle. A slower lifestyle allowed for “*more time together as a family” (Participant 16)* which resulted in more family bonding activities such as board games, bible study and increased connection amongst family members. Additionally, with all members of the family being impacted by a slower pace, parents working from home noted a more positive environment. A decreased pace in lifestyle also had psychological benefits. Respondents appreciated not being “*dictated by a pre-set schedule” (Participant 9)*. They also saw an improvement in their child's “*mood…attention and energy” (Participant 4)* and they were able to sleep in the mornings and enjoy more free time.

Another frequently conveyed positive outcome amongst participants was the improvement of health outcomes among their children and families. Respondents reported that, contrary to previous seasons, they weren't recurrently symptomatic for “*any type of cold or contagious illness since the pandemic began*” (*Participant 19)*. This was attributed to significantly decreased social interaction during the lockdowns and school shutdowns, increased handwashing, and adopting face coverings as common practice. For families with chronically ill children, decreased illness meant more than just overall better health. One participant noted that when her child “*gets sick, she gets really sick and typically ends up in hospital*” *(Participant 15)*. Another respondent indicated that health improvements resulted in them being able to cancel a previously scheduled surgery “*to place [tympanostomy] tubes…as ears were clear” (Participant 19)*. Many responses echoed the positive impact that fewer hospital visits had on their families due to the transition of many medical appointments to virtual delivery. Virtual care was suggested as further contributing to a slower-paced life, thus having a positive impact on family scheduling logistics.

### Theme 2: negative effects of COVID-19 on the families of children using NIV

Negative outcomes related to the experience of children using NIV during the COVID-19 pandemic were also reported (Table 3), “*like the rest of us whether we are adults or kids, the pandemic has impacted our lives physically and emotionally” (Participant 47)*. The most frequent response was related to increased stress and uncertainty for both the child and their family. Respondents used words like scary, afraid, concerned, fear, and anxiety, to describe feelings of health-related stress that were prevalent, as their children were already ill before the pandemic began. Many families reported heightened concern stemming from the reality of having kids with “*health issues and autoimmune disorders*” *(Participant 27)*. One parent reported that “*due to our daughter being extremely high risk we have to be a lot more cautious and careful with the things we do and the people we allow around her” (Participant 15)*. This was a common feeling among respondents who felt an increased sense of stress regarding the possibility of their child catching the COVID-19 virus and developing severe COVID-19 respiratory disease. In contrast, there was no mention of grief due to severe disease or the death of family members or friends. Stress also stemmed from a general uncertainty, with parents noting that “*nobody knows where this is heading*” *(Participant 34)* and fear of “bringing *the virus back into our house from work.”* The added stress of misinformation and the challenge of “*sorting out truth versus what we are told to believ*e” (*Participant 4)* was also reflected in the survey responses.

**Table 3:** Data excerpts for theme “negative effects of COVID-19 pandemic on child and family.”

“*We miss our family and friends, and it has affected one of my children more who already had social issues prior to the pandemic. Fears are that one of us might catch the virus and spread it to my child who has lung disease. Other stressors are helping the kids do online schooling while also working from home and taking care of household duties.”*—Participant 5
“*Financials have been hard especially when replacement tubes and masks are expensive. I'm a single mom to 4, 3 autistic and have a CPAP as well but can't afford to replace anything so I make do. I work from home in a salon, and I have been shut down 3 times and I work a retail job and go to school all while home schooling my kids so it's been hard.”*—Participant 48
“*Frustration about the situation and how his work life hadn't changed and how I was expected to do my job, look after children and somehow keep the house clean while he was working.”*—Participant 17
“*Stressors, fear of illness related to her airway implications. Financial losses and increased bills are causing burden, all specialists are a travel min 5 hours for us. My daughter takes forever to fall asleep, stay in her bed, stay asleep, wakes tired and cranky since this all played out.”*—Participant 49

Other emerging health-related negative outcomes included weight gain and sedentarism. Parents attributed this to their children's lack of exercise and physical activity as school, sports, and extracurricular activities were canceled.

Social isolation during lockdowns was a recurrent comment described as having a significant impact on their children's mental health, with children “*missing their friends*” *(Participant 51)*, extended family members and other community-based groups such as church. Many participants had to choose online/home-schooling for their children or opted for it as an extra precaution when school shutdowns were lifted, which further increased their children's isolation. Concerns about their children's academic performance in an online environment were also expressed by several participants. Some parents noted a negative change in their own physical and mental health and their relationship with their partners.

Participants perceived a lack of access to certain healthcare resources resulting from the pandemic, including concerns regarding the lack of pediatric services available due to allied health professionals being redeployed for an emergent COVID-19 response. Limited supplies of health equipment related to NIV were also highlighted as a negative effect of the COVID-19 pandemic, specifically distilled water was often sold out in stores.

Workday losses and financial stress were explicitly mentioned as having an overall negative impact on families and specifically on keeping up with the NIV equipment, with one respondent sharing they were unable to afford replacement NIV tubes and masks throughout the COVID-19 pandemic. Work-home gender imbalances were reflected as a source of frustration: “*his work life hadn't changed while I was expected to do my job, look after children and somehow keep the house clean while he was working.”*

### Theme 3: neutral effects of COVID-19 on the families of children using NIV

While most responses highlighted positive or negative implications, some participants simply reported the COVID-19 pandemic resulted in a change of routine (Table 4), with limited detail. One participant noted that many healthcare appointments were “*limited to virtual appointments” (Participant 50)* implying a negative limitation, however, they continued to say “*But this works for us as we are 2 hours away,”* implying a positive benefit. Another participant reflected on their decision to postpone check-ups except for the essential ones. One response described changes that a child using NIV adapted to including “*wearing a mask, social distancing, and* [using] *hand sanitizer frequently” (Participant 45)*.

**Table 4:** Data excerpts for theme “neutral effects (new routines) of COVID-19 pandemic on child and family.”

“*We have also postponed most check-ups (except pediatricians) or opted for virtual check-ups/appointments when possible.”*—Participant 14
“*My child started to use the CPAP machine right when the pandemic started in March 2020. He attended school in person and had to adjust to wearing a mask, social distancing, and using hand sanitizer frequently.”*—Participant 45
“*We are limited to virtual appointments but works for us as we are 2 hrs away”*—Participant 50

### Theme 4: impact of COVID-19 on NIV adherence

**Figure 2:**
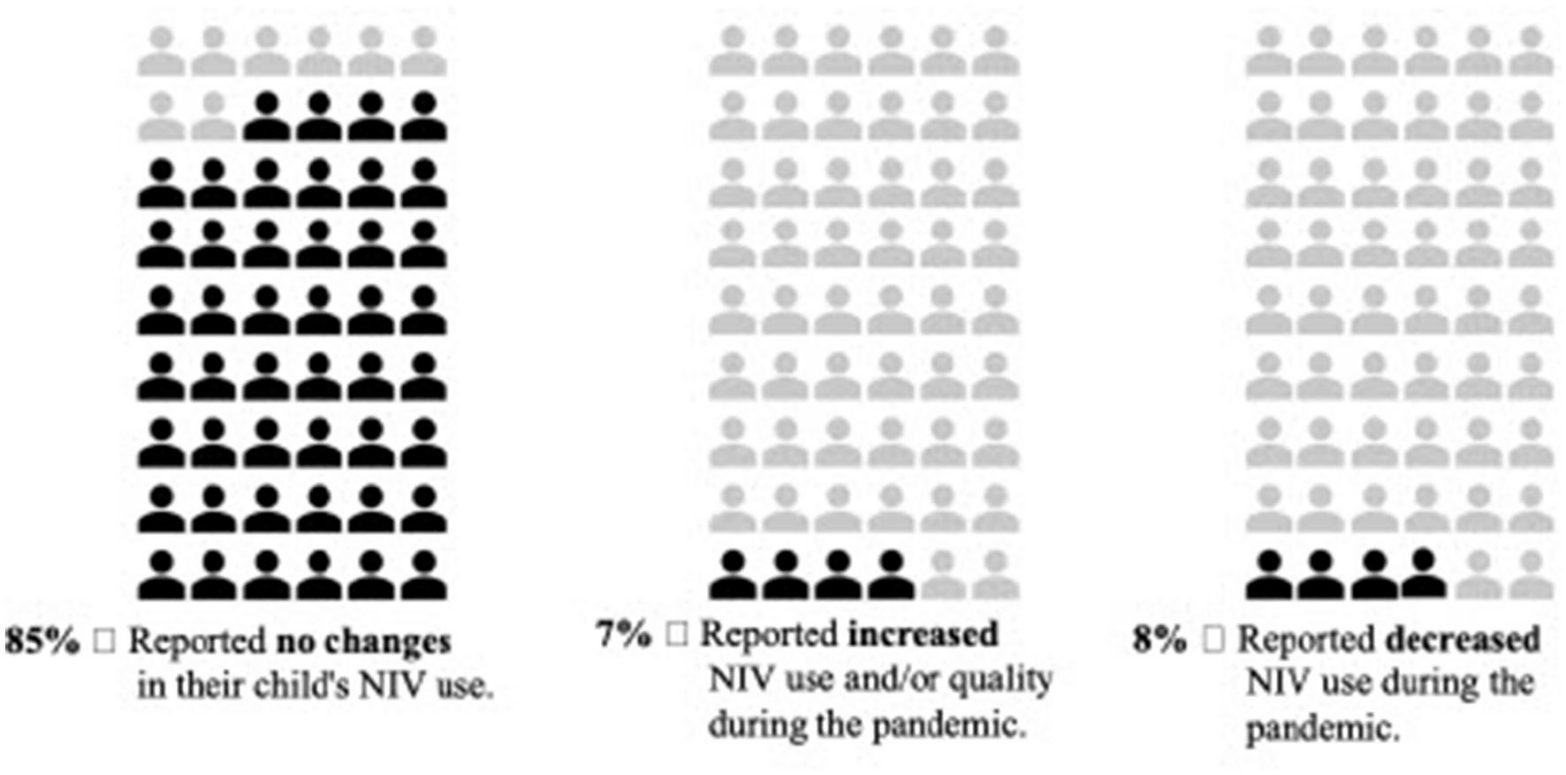
Self-reported NIV adherence during COVID-19.

When asked how COVID-19 impacted their children's adherence to NIV, there was overwhelming perception of their child maintaining similar NIV use than pre-pandemic in 85% of respondents, despite the challenges and life changes associated with the COVID-19 pandemic (Figure 2). Some participants, however, pointed out the need for more incentives and conscious discipline to maintain NIV use.

Seven percent of participants perceived their child was able to increase their NIV use during the COVID-19 pandemic. Parents attributed the rise in use to longer sleep times coupled with more parental presence at bedtime resulting in more consistency of their child's sleep routine. For example, one participant reported “*Online schooling has allowed more time in the mornings to rest on BPAP before having to get up” (Participant 23)*.

A small proportion of participants (6%) reported their child decreased their NIV use (Table 5). Noted reasons for decreased NIV adherence included parenting challenges or difficulty accessing the NIV resources and supplies, particularly distilled water.

**Table 5:** Data excerpts for caregiver-perceived changes in their child's NIV adherence during COVID-19 pandemic.

“*I haven't been as diligent as a parent and a bit too forgiving because of the summer break and working from home. I've only in the last two weeks really put my foot down and my child feels so much better when he wears his CPAP at night which makes him want to wear it.”*—Participant 1
“*Was a bit harder to get distilled water and replacement parts for the machine and mask. More consistent and better use as he has not been sick.”*—Participant 19
“*Online schooling has allowed more time in the mornings to rest on BPAP before having to get up.”*—Participant 23
“*Not really. We have a solid home care routine and schedule. It was built to navigate both medical and life challenges. It's evident that this established care routine for my child also works during the pandemic.”*—Participant 44

## Discussion

This paper adds new insights into the drastic changes that COVID-19 has caused in the lives of children using NIV and their families. Unexpectedly, this demographic experienced similar positive changes to other pediatric populations such as increased family time, slower lifestyles, and less acute respiratory illness. However, there was a perceived higher risk and vulnerability for children with chronic respiratory conditions using NIV, resulting in amplified negative effects of the pandemic in this population. Families of children using NIV experienced even more and longer isolation, fear, and anxiety, on top of difficulties finding supplies to maintain NIV equipment or accessing other health-related supports. Despite significant changes in their overall lives, families perceived no changes or even improved children's adherence to NIV through the first year of the pandemic. Further, the drastic changes in the healthcare system to virtual care were mostly perceived as advantageous to children and families.

The themes extrapolated from our data support results from previous studies on the social implications of COVID-19 in other pediatric populations. Many of the negative impacts experienced by children using NIV and their families paralleled those described in the general population, including increased symptoms of anxiety, depression, and decreased sleep quality amongst the pediatric population and caregivers (Cahal et al., 2021; Cardenal-Munoz et al., 2021; El-Osta et al., 2021; Meherali et al., 2021). However, parents of children using NIV conveyed sustained levels of high anxiety related to the possibility of their chronically ill child contracting COVID-19. Caregivers' fears at that time were likely well-founded given the level of uncertainty, the lack of a COVID-19 vaccine available for pediatric ages, and the high level of complexity of our study cohort, as demonstrated in our previous quantitative analysis (Halperin et al., 2023). Early pandemic studies in patients with chronic illnesses such as chronic lung or neurological diseases, and frequent home NIV users, have reported increased risk for severe COVID-19 disease requiring hospitalization and even intensive care (Cahal et al., 2021; Di Cicco et al., 2021). Interestingly, other negative effects reported in the literature such as increased screen use and shorter sleep times were not mentioned in our study (Carson et al., 2019). Our study was novel at demonstrating that the negative impacts of the pandemic were significantly amplified by family's decisions to limit in-person social interactions even longer, beyond the periods of imposed lockdown, like previous literature in other chronically ill populations (Alaqeel et al., 2021; Beytout et al., 2021; Masi et al., 2021). The long-term effects post-pandemic of this prolonged isolation in children who already face challenges to integrate in their communities, however, are unknown. In addition, our study confirmed that child caregiver responsibilities continue to burden female parents to a larger degree, perpetuating known social gender disparities. This information can help inform government measures to protect the most vulnerable during future pandemics while developing school-based infrastructures and programs that facilitate/promote social connection, peer support, and daily exercise even in the light of physical distance, as well as government-supported strategies toward work/home balance that address the gender bias.

These study results reflect the impact of misinformation on vulnerable populations. This is important as previous research has shown higher vaccination hesitancy because of misinformation, with the subsequent risk for adverse outcomes (Capurro et al., 2022; Kim et al., 2022; Ruiz and Bell, 2022). Further advocacy work is required to identify effective strategies that protect vulnerable populations against misinformation.

Overall, there was a parental perception of a maintained child's adherence to NIV during the COVID-19 pandemic. If anything, the pandemic allowed for lifestyle changes that promoted more parental support and a consistent sleep schedule that might have contributed to adequate adherence. It is also possible that the rapid change to virtual care would have allowed for enough child/family support and contributed to adequate maintenance of child adherence perceived by caregivers. This was confirmed by our quantitative study analyzing the machine download data showing overall adequate adherence in most of the child home NIV users in our center (Halperin et al., 2023). These findings along with previous quantitative data suggest that families in our center may prioritize strategies to maintain adequate NIV use despite the challenges faced during the COVID-19 pandemic. Interestingly, there was no specific mention of parental fear of respiratory disease as a drive to maintain NIV adherence, as suggested in previous adult studies (Attias et al., 2020; Batool-Anwar et al., 2020).

Finally, there was a clear reference to the benefits of shifting toward a virtual healthcare system tailored to patients requiring recurrent encounters with the health system such as children using home NIV, including accessibility of the team, virtual equipment assessment and use of iCloud-based machine downloads. This was found in previous research reporting benefits from virtual care including fewer trips to the hospital, less missed school time, and less exposure to respiratory illness (Esposito et al., 2020; Paruthi, 2020; Hoi et al., 2021; Nobili et al., 2021; Taylor et al., 2021). Despite the challenges associated with a rapid change to virtual care and limited access to certain health-related services, families felt well supported by the NIV program virtually through the first year of the COVID-19 pandemic. These findings provide an opportunity for learning and reflection on how we can optimize the provision of health care in this population going forward. More studies are needed exploring efficient ways to provide virtual medical care on patients using medical technology such NIV therapies.

This survey was designed to explore how the lives of children using home NIV were affected by the pandemic and whether the pandemic had repercussions on their NIV use. Therefore, it was out of the scope of this study to explore overall barriers to NIV adherence. There may be a positive reporting bias toward participating caregivers of who are invested in their children's wellbeing and fostering high adherence to NIV. This might limit the interpretation of certain study findings such as the pandemic's impact on children who struggle with NIV adherence to begin with. To mitigate this risk, this survey was offered anonymously to all caregivers attending the clinic, providing an opportunity for an honest response.

## Conclusion

The COVID-19 pandemic had significant implications for children with chronic respiratory disease using NIV. While the positive effects of the pandemic echoed findings from studies in general pediatric populations, the negative effects were more profound including their fear of severe disease in their child with already chronic respiratory disease and the profound isolation that was prolonged beyond the mandatory pandemic restrictions, as well as gender disparities between caregivers' responsibilities and its toll in mental health. Overall, caregivers prioritized NIV use by their children despite the challenges and recognized the distinct benefits of the virtual model of specialized care established during a pandemic. Lessons learned during the first year of living under the COVID-19 pandemic have the potential to inform meaningful changes in the provision of health care.

## Data availability statement

The original contributions presented in the study are included in the article/supplementary material, further inquiries can be directed to the corresponding author.

## Ethics statement

The studies involving humans were approved by the Health Research Ethics Board at the University of Alberta. The studies were conducted in accordance with the local legislation and institutional requirements. The participants provided their written informed consent to participate in this study. Written informed consent was obtained from the individual(s) for the publication of any potentially identifiable images or data included in this article.

## Author contributions

LD: Formal analysis, Writing—original draft, Writing—review & editing, Validation. EM: Formal analysis, Writing—original draft, Writing—review & editing, Validation. HH: Writing—review & editing, Validation. DO: Supervision, Writing—review & editing, Conceptualization, Investigation, Methodology. SS: Supervision, Writing—review & editing, Conceptualization, Investigation, Methodology. MC-C: Conceptualization, Data curation, Funding acquisition, Investigation, Methodology, Project administration, Resources, Software, Supervision, Validation, Visualization, Writing—original draft, Writing—review & editing.
